# Magnetism and magnetoresistance of single Ni–Cu alloy nanowires

**DOI:** 10.3762/bjnano.9.219

**Published:** 2018-08-30

**Authors:** Andreea Costas, Camelia Florica, Elena Matei, Maria Eugenia Toimil-Molares, Ionel Stavarache, Andrei Kuncser, Victor Kuncser, Ionut Enculescu

**Affiliations:** 1 National Institute of Materials Physics, PO Box MG-7, 077125, Magurele-Bucharest, Romania; 2 GSI, Helmholtz Centre, Planck str. 1, D-64291, Darmstadt, Germany; 3 University of Bucharest, Faculty of Physics, PO Box MG-11, 077125, Magurele-Bucharest, Romania

**Keywords:** nanowires, magnetic properties, magnetoresistance

## Abstract

Arrays of magnetic Ni–Cu alloy nanowires with different compositions were prepared by a template-replication technique using electrochemical deposition into polycarbonate nanoporous membranes. Photolithography was employed for obtaining interdigitated metallic electrode systems of Ti/Au onto SiO_2_/Si substrates and subsequent electron beam lithography was used for contacting single nanowires in order to investigate their galvano-magnetic properties. The results of the magnetoresistance measurements made on single Ni–Cu alloy nanowires of different compositions have been reported and discussed in detail. A direct methodology for transforming the magnetoresistance data into the corresponding magnetic hysteresis loops was proposed, opening new possibilities for an easy magnetic investigation of single magnetic nanowires in the peculiar cases of Stoner–Wohlfarth-like magnetization reversal mechanisms. The magnetic parameters of single Ni–Cu nanowires of different Ni content have been estimated and discussed by the interpretation of the as derived magnetic hysteresis loops via micromagnetic modeling. It has been theoretically proven that the proposed methodology can be applied over a large range of nanowire diameters if the measurement geometry is suitably chosen.

## Introduction

Nowadays, due to the continuous search for new electronic devices that exploit specific properties of nanostructures, the methods of controlled fabrication are in the spotlight of researchers. Metallic magnetic nanowires are a particular class of nanoobjects that can be used in a wide range of applications such as magnetoresistive sensors and data-storage elements or spintronic devices [1]. Their specific magnetic and magneto-transport properties, directly related to their easily tunable composition, structure and morphology, are making them perfect candidates for such novel devices [1–2]. For example, in contrast to other nanosized magnetic systems, the nanowires exhibit an additional degree of freedom associated to their inherent shape anisotropy, depending on the aspect ratio (the ratio between the length of the wire and its diameter). Both the aspect ratio and the wire diameter influence the magnetic domain structure of the wire, which is also related to an exchange length parameter, λ_ex_ = π√(*A*/*K*), where *A* is the exchange stiffness and *K* is the anisotropy constant, respectively. Even for single-component metallic nanowires (e.g., Ni) of high aspect ratio, different magnetization reversal mechanisms might be in work (from the simplest coherent such as rotation to the more complex transversal and vortex wall modes), depending on the wire diameter [2–5]. Accordingly, the associated magnetoresistive phenomena due to scattering of conduction electrons on different field-dependent spin configurations can be also tuned. The magnetic and associated magnetoresistive properties of nanowires can be further modified in multiple segment nanowires consisting of ferromagnetic (e.g., Ni) and nonmagnetic (e.g., Cu) successive segments making such systems interesting for spintronic applications [6–7].

Another possibility to control the scattering of conduction electrons on magnetic configurations is through composition-dependent magnetic properties in binary or ternary alloy nanowires. Efforts in obtaining multilayered nanowires with optimized giant magnetoresistance (GMR) effects by changing the composition of the ferromagnetic segments (e.g., Co–Ni–Cu, Co–Ni) have been reported in the last two decades [8–9]. Characteristic to these magnetoresistance studies is that the GMR effects were measured in currents perpendicular to the plane geometry on arrays of nanowires in different membranes, namely with current flowing perpendicular to the membrane (along the wires) and with the field applied along the membrane plane (perpendicular to the wires). The thickness of the segments (either conductive or ferromagnetic) was of a few nanometers, involving monodomain-like configurations and possible exchange couplings of magnetic segments specific to GMR systems.

To date, less interest has been shown for the magnetoresistive properties of overall alloy nanowires, where magnetic parameters influencing the magnetization reversal (e.g., *A*, *K* or the local value of the spontaneous magnetization) and hence the scattering of the conduction electrons can be easily tuned via the nanowire composition. However, an anisotropy magneto-resistance (AMR) mechanism would be expected for such systems instead of GMR. As a direct consequence, this much weaker effect can no longer be detected in arrays of nanowires but only in single nanowires. Magneto-resistance and magneto-thermopower measurements on single Co–Ni alloy nanowires have been reported [10].

Several methods have been reported for the controlled fabrication of magnetic nanowires including lithographical techniques, chemical vapor deposition and hydrothermal growth [11–13]. The main purpose of all these specific methods is to have a good and simultaneous control of morphology, structure and composition in order to succeed in tailoring the magnetic properties of the nanowires.

A very useful technique to fabricate nanowire arrays is electrochemical deposition inside nanoporous membranes [14–20]. Some of the advantages of this method are low cost, high reproducibility and fabrication at low temperatures. By using this approach to obtain nanowires, one can control both their morphology (by means of the geometry of the template) and their composition. Consequently, the magnetic properties of such nanowires can be tuned in agreement with the foreseen applications. The templates used are usually nanoporous ion-track polycarbonate membranes [21] and anodic alumina membranes [22].

Both Ni–Cu alloys and Ni–Cu multilayers have been intensively studied as simple binary systems in several dimensionalities, i.e., the bulk form, thin films and nanowires. Single Ni–Cu alloy nanowires might be seen as case study for a unidimensional soft-magnetic system with arrays of such unidimensional systems attracting wide interest [23–25]. The advantage of the single ferromagnetic Ni–Cu alloy nanowire is the simplicity of tuning magnetic parameters through composition and morphology of the nanowire as well as the suitability for micromagnetic modeling of the magnetic behavior. However, the main disadvantage is the difficult experimental magnetic characterization due to the extremely low associated magnetic moment needing peculiar experimental configurations and specific ultra-sensitive magnetic sensors such as micro-SQUID (superconducting quantum interference device) detectors [26]. Arrays of such nanowires (thousands of single elements) can be investigated by usual SQUID magnetometers, but the overall magnetic behavior of the assembly cannot be straightforwardly connected to the magnetic behavior of the single nanowire. It depends not only on the size dispersion of the nanowires but also on their geometrical configuration and mutual magnetic interaction among the single magnetic elements, usually of dipolar type.

In this paper we report on a strategy of studying the magnetic properties of single Ni–Cu alloy nanowires of high aspect ratio via specific anisotropic magnetoresistance measurements. In this respect, arrays of nanowires have been fabricated by means of templated electrochemical replication, using as templates chemically etched polycarbonate membranes irradiated with swift heavy ions. Individual Ni–Cu alloy nanowires of different compositions have been contacted on interdigitated metallic electrodes by using electron beam lithography (EBL) and magnetoresistive measurements have been performed. It has been proven by micromagnetic calculations that Stoner–Wohlfarth-like magnetization reversal mechanisms are still active in the perpendicular geometry of these systems up to nanowire diameters of 100 nm. Therefore, a direct simple methodology for transforming the magneto-resistance data in corresponding magnetic hysteresis loops can be applied, opening the way for an easier experimental investigation of magnetization reversal in ferromagnetic single nanowires. Information about magnetic parameters and phase segregation in single nanowires has been obtained by exploiting the magnetic hysteresis loops derived from magnetoresistance measurements and the associated micromagnetic modeling.

## Results and Discussion

SEM micrographs of arrays of Ni–Cu alloy nanowires show that the nanowires obtained at all four different electrodeposition potentials have a regular cylindrical shape with smooth walls along their entire 30 μm length (corresponding to the polymer membranes thickness) and an average diameter of about 100 nm (SEM images for arrays of Ni–Cu alloy nanowires obtained for −1000 mV are presented in Figure S1, Supporting Information File 1.

**Figure 1:**
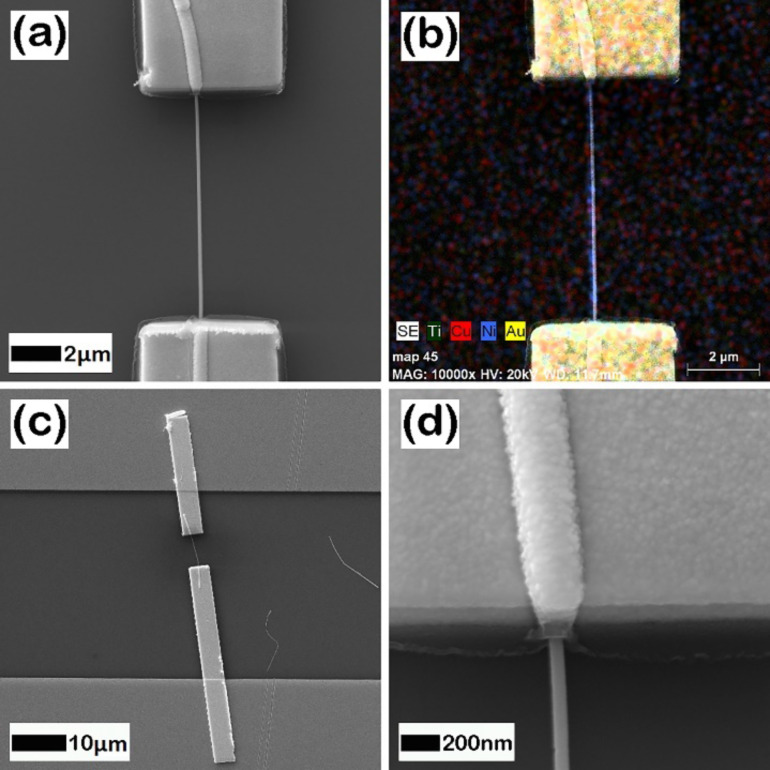
(a, c, d) SEM images (at different magnifications) of a Ni–Cu alloy nanowire grown electrochemically and contacted by EBL; (b) EDX analysis of the distribution of elements in the sample (Ti/Au and Ni–Cu).

SEM images recorded at different magnifications for a typical individually contacted Ni–Cu alloy nanowire are displayed in Figure 1 and Figure S2, Supporting Information File 1. The composition of the Ti/Au electrodes (100/200 nm) and single Ni–Cu alloy nanowire was confirmed by using EDX analysis (Figure 1b and Figure S3, Supporting Information File 1). The compositions of the four types of nanowires, corresponding to the four different electrodeposition potentials, as more precisely obtained by the EDX analysis performed on bunches of nanowires, are 20, 54, 75 and 92 atom % of Ni. Also, the saturation magnetization of the corresponding nanowires can be estimated from typical SQUID magnetometry measurements performed on arrays of nanowires, as reported in [27]. These four samples of single Ni*_x_*Cu_1−_*_x_* alloy magnetic nanowires of different compositions of Ni (0.2 < *x* < 0.92) and with diameters of about 100 nm (see Figure 1d), contacted as described above, have been further considered for the magnetoresistance measurements.

**Figure 2:**
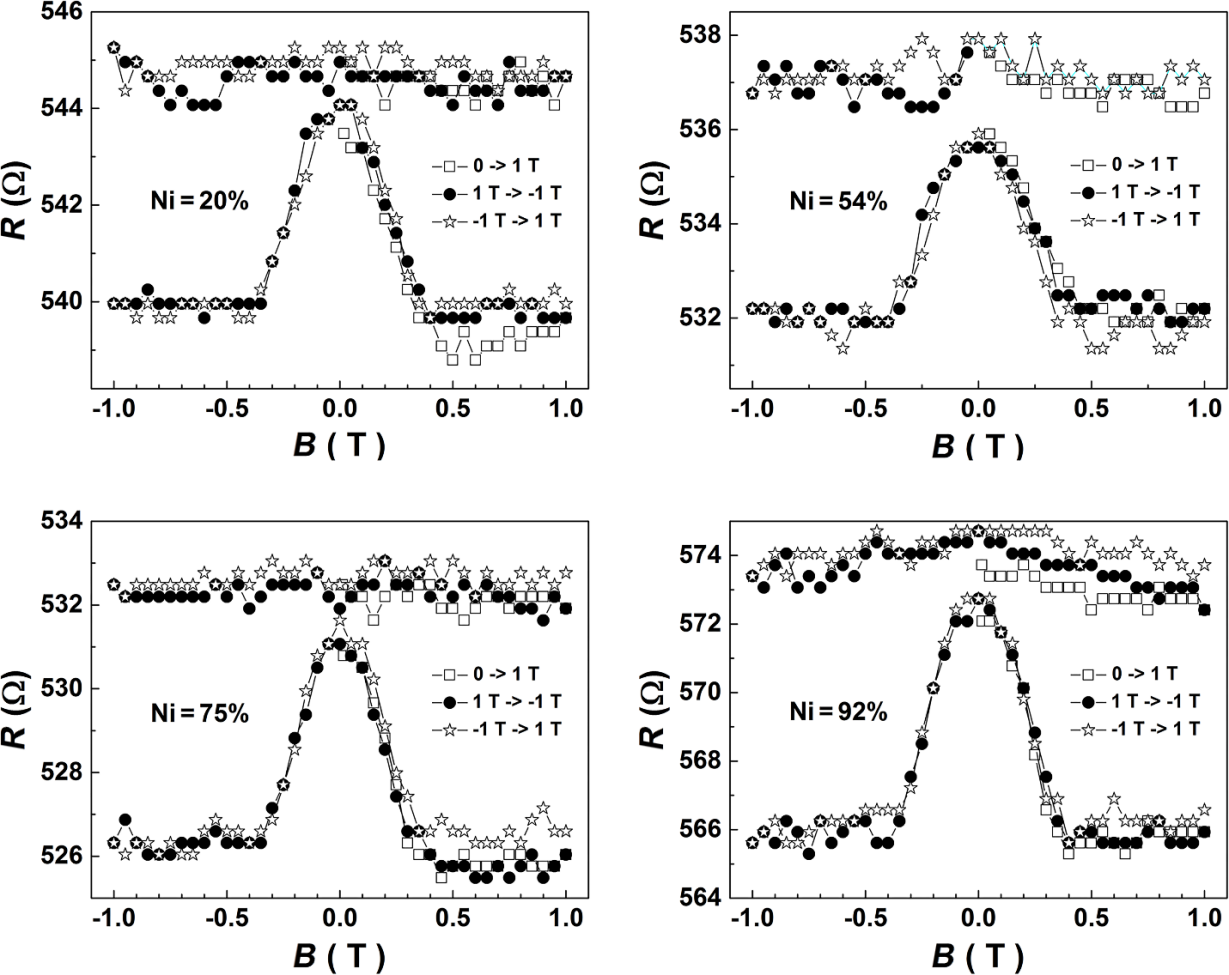
Magnetoresistance measurements obtained on the four single Ni–Cu alloy nanowires analyzed (corresponding to different concentrations of Ni), in both parallel (flat upper curves) and perpendicular (peaked lower curves) geometry. With no field applied, the magnetization lies along the wire due to the strong shape anisotropy.

Typical magnetoresistance measurements obtained from the four samples coded as SNW0 (*x* = 0.2), SNW1 (*x* = 0.54), SNW2 (*x* = 0.75) and SNW3 (*x* = 0.92) are shown in Figure 2. As a general observation, for all of the samples, one observes the lack of any magnetoresistive effect in the parallel geometry and a magnetoresistive effect of the order of percent in the perpendicular geometry. Such behavior clearly reminds on anisotropic magnetoresistance effects the physical mechanisms of which were excellently described already by McGuire and Potter in 1975 [28]. Accordingly, in a magnetic monodomain system, the resistivity depends on the orientation of the spontaneous magnetization with respect to the current density. If θ is the angle between the magnetization and the current, the resistivity ρ(θ), can be expressed as:

[1]
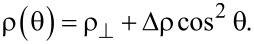


It is evident that if the field is perpendicular to the direction of the current (cos^2^ θ = 0), a perpendicular resistivity is measured, 
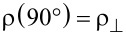
, whereas if the field is parallel to the current direction (cos^2^ θ = 1), a parallel resistivity is measured, 
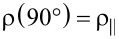
, with 
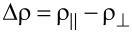
.

In a magnetic monodomain with uniaxial anisotropy, the magnetization reversal canb be described using the Stoner–Wohlfarth model [29–30]. Thus, if the field is applied along the easy axis (EA), the magnetization changes steeply its orientation along the EA at a switching field proportional to the anisotropy constant and inversely proportional to the spontaneous magnetization. When the current flows also along the EA, a sharp reversal of the localized magnetic moments from the current direction (θ = 0°) to the opposite direction (θ = 180°) has no influence on the resistivity (cos^2^ θ = 1) and hence the parallel resistivity, 
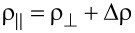
, should remain constant with the field, even in case of such a drastic change of the magnetic configuration (see analogy with Figure 2). When the field is applied perpendicular to the EA (e.g., decreasing from a value above the saturation field), a gradual reorientation of the magnetic moments is expected, leading to a linear decrease of the magnetization from positive to negative saturation, with the saturation field equating the switching field in parallel geometry. If the current flows along the EA, the resistivity is 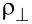 above the saturation field (θ = 90°) and 
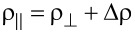
 at zero field (where the magnetization lies along the EA, due to the shape anisotropy). It is worth mentioning that Δρ might be positive or negative depending on whether the electron conduction is dominant by spin-up or spin-down electron scattering as well as by the ratio between the spin–orbit coupling parameter and the splitting of the uppermost bands of the compound, being therefore material-dependent.

However, in most of the Ni alloys, including Ni–Cu, Δρ is positive, meaning electrons flowing along the magnetic moments are scattered strongest (see analogy with Figure 2). Once the anisotropy of the resistivity with respect to the magnetic configuration is introduced and the resistance of wires with similar geometric characteristics is experimentally measured, the magnetoresistance of the wires can be easy introduced as: *MR* = [*R*(*B*) − *R*(∞)]/*R*(∞), taking values between 0 and *MR*^max^ = [*R*(0) − *R*(∞)]/*R*(∞). While the magnetic moments are expected to be aligned along the wire axis (EA) at zero field (after saturation) due to the shape anisotropy, *MR*^max^ in parallel geometry has to be 0 (as experimentally confirmed) whereas in perpendicular geometry 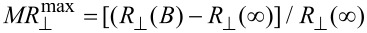.

**Figure 3:**
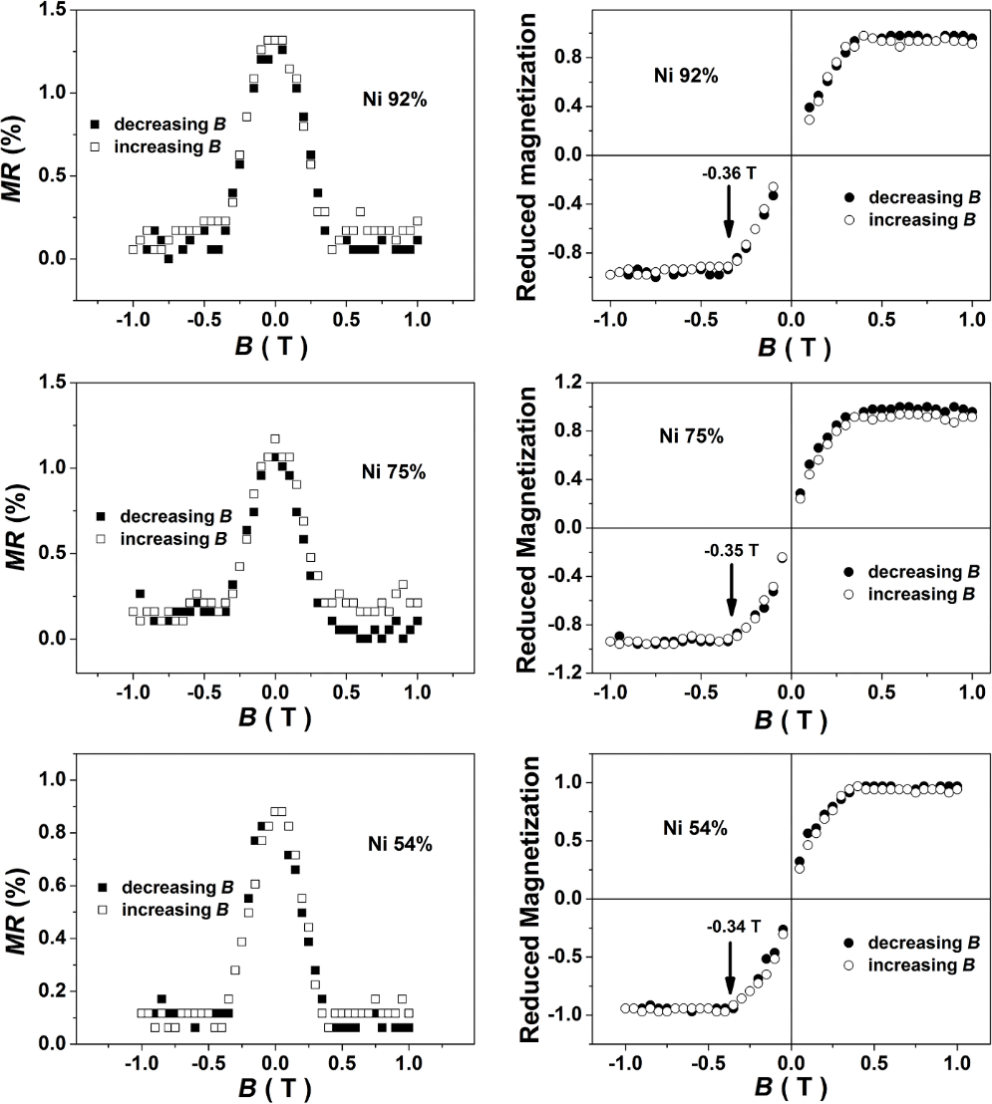
The perpendicular magnetoresistance as a function of the induction of the magnetic field (left column) and the corresponding magnetization reversal in perpendicular geometry (right column) as calculated via Equation 2, for single Ni–Cu alloy nanowires of different Ni concentrations.

The dependence of the perpendicular magnetoresistance on the applied field in three nanowires SNW1, SNW2 and SNW3 is shown in Figure 3. SNW0 was left out because it belongs to the sample with the highest Cu content, where a strong segregation was already confirmed in [27].

Assuming Stoner–Wohlfarth-type magnetic behavior, at least in the case of perpendicular geometry (where magnetoresistance effects are present), the magnetization reversal can be obtained by starting from the magnetoresistance data, via Equation 1 written in terms of resistances and considering the projection of the spontaneous magnetization along the field direction as *M*_S_ = sin θ. Accordingly, from Equation 1 applied for perpendicular geometry follows:

[3]
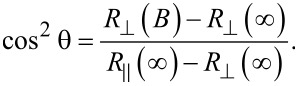


While at zero applied field the magnetic moments are assumed to be oriented along the wire axis due to the shape anisotropy, 
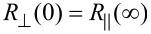
, under the condition that the field is directed entirely along the EA (wire axis). Therefore, cos^2^ θ at *B* = 0 T should be 1. Any deviation of the field orientation from the EA will lead to a value smaller than one for cos^2^ θ, the deviation angle θ_0_ being provided by the experimental ratio:

[4]
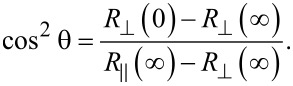


Having in mind the expression of the magnetoresistance 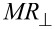, Equation 3 can be re-written:

[5]
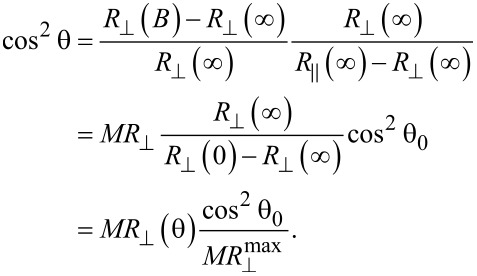


The relative magnetization in perpendicular geometry can be expressed by:

[2]
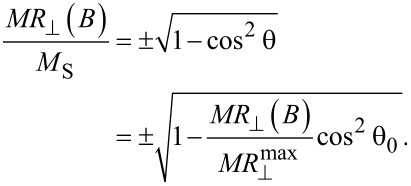


The reduced magnetization in perpendicular geometry as a function of the applied field, deduced from Equation 2 using the experimental values of the magnetoresistance, are shown in the right column of Figure 3. The very slow variation of 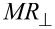 for field values close to 0 (even below the experimental error) makes the correct evaluation of the magnetization in very low fields unrealistic. Values for θ_0_ the range from 10° to 15° were estimated for all samples via Equation 4.

All the derived loops presented in the right column of Figure 3 resemble a Stoner–Wohlfarth (S–W)-type magnetization reversal with the field applied perpendicular to the easy axis. The saturation field (approaching the switching field in case of the S–W model) decreases very slowly with the Ni composition, contrary to what would be expected from the composition dependence of the saturation magnetization and inverse proportionality of the switching field with the saturation magnetization. Therefore, before discussing the specific aspects of the magnetic reversal mechanisms accompanying the above mentioned magnetoresistive behavior, micromagnetic simulations on this type of nanowires will be considered in detail.

**Figure 4:**
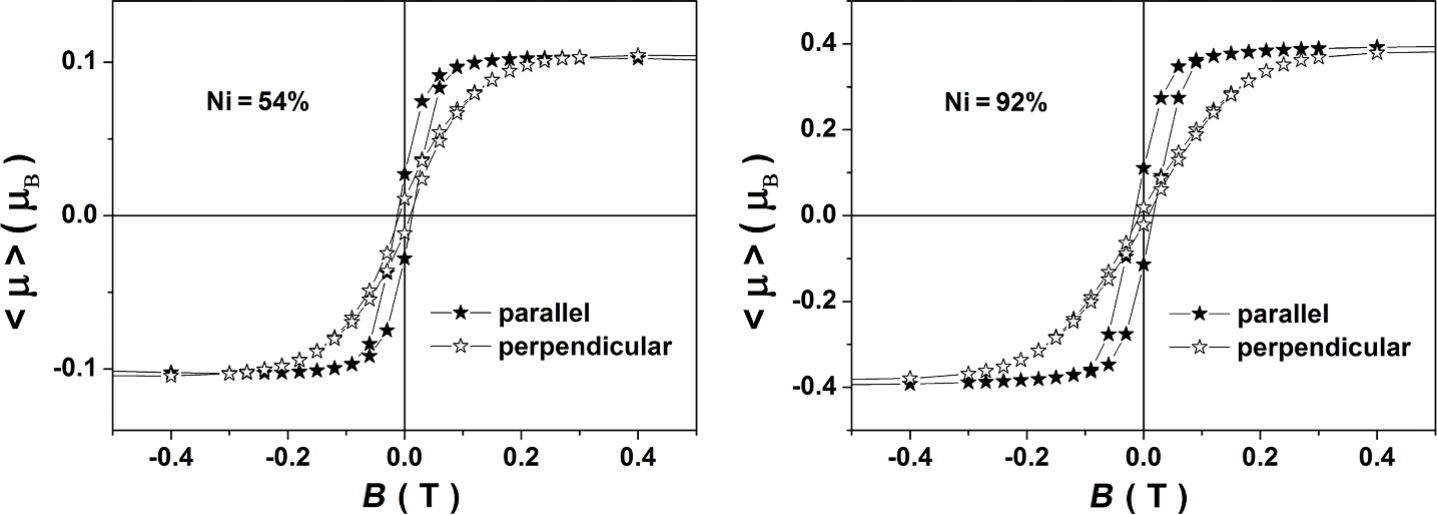
Hysteresis loops showing the average magnetic moment of Ni atoms as a function of the applied magnetic field, obtained from SQUID magnetometry measurements at 300 K on arrays of Ni–Cu nanowires of different compositions (54 and 92 atom % of Ni) with the field applied parallel and perpendicular to the nanowires.

Nanowires with high aspect ratio (1:10) have been chosen for the simulations to optimize the computing time. No meaningful differences in the magnetic reversal process are expected for higher aspect ratios because of the dominant shape anisotropy. For the same reason of reduced computing time, the initial estimations have been performed with nanowires of diameter lower than that of the experimental samples. Thus, nanowires of 400 nm length and 40 nm in diameter have been meshed in small 3 nm × 3 nm × 4 nm tetragonal cells. The magnetization reversal under an applied field ranging from −1.00 T to 1.00 T was calculated. The field was applied either along the wire (providing the parallel magnetization reversal) or perpendicular to it (providing the perpendicular magnetization reversals), and at low deviations (10° or 15°) from the reference axes. All simulations were time-independent and magnetostatic energy minimizations via the conjugate gradient method were performed for all points obtained on the hysteresis plot. In addition to the abovementioned geometrical aspects, the simulations require also two additional magnetic parameters: (i) the spontaneous magnetization (well approximated by the saturation magnetization) and (ii) the stiffness constant (the magnetocrystalline anisotropy constant of the material is proven to be less relevant in structures where the shape anisotropy is dominant). To provide realistic values for *M*_S_ under similar conditions to the magnetoresistance measurements, hysteresis loops at 300 K were collected by SQUID magnetometry on arrays of nanowires in polycarbonate matrices with the applied field oriented both along and perpendicular to the nanowires. After subtracting the diamagnetic contribution and applying the same method to estimate the amount of the measured magnetic material as in [27], hysteresis loops involving the field dependence of the average magnetic moment per formula unit were obtained (as exemplified in Figure 4 for the case of samples with 54 and 92 atom % of Ni). Average magnetic moments per Ni atom of 0.38µ_B_, 0.22µ_B_ and 0.12µ_B_ (corresponding to *M*_S_ values of 3.0·10^5^, 1.7·10^5^ and 0.9·10^5^ A·m^−1^) were obtained for samples with 92, 75 and 54 atom % of Ni, respectively.

**Figure 5:**
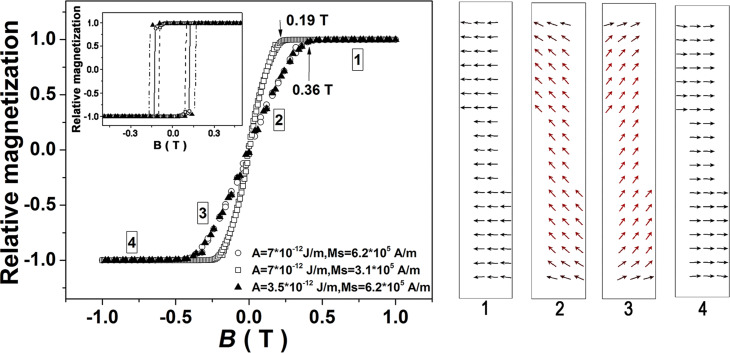
Magnetization reversal in almost perpendicular geometry at two different values of saturation magnetization and stiffness constant. The corresponding magnetization reversals in almost parallel geometry are shown in the inset. A deviation of the wire axis from the orthogonal axis of about 10° was considered. The evolution of the spin structure at four different decreasing field values is graphically presented on the right-hand side of the figure (the positive field is oriented almost perpendicular to the wire toward the left side).

These values are much lower than the theoretical values corresponding to the given Ni concentrations [31]. This is most probably due to a lower density of filled pores connected to evolution of hydrogen bubbles at the electrode [27]. However, the above estimations allowed us to perform micromagnetic simulations on similar cylindrical magnetic structures of high aspect ratio with saturation magnetizations of about 10^5^ A·m^−1^ and stiffness constants of about 10^−22^ J·m^−1^ (as specific to Ni and Ni alloys) [31–32]. The magnetization reversal values obtained in perpendicular geometry for two values of saturation magnetization and stiffness constant are shown in Figure 5 (the inset of the same figure presents the magnetization reversal process in parallel geometry).

According to the simulations, the saturation field in perpendicular geometry is almost insensitive to values of the stiffness constant, but decreases strongly with the saturation magnetization. The evolution of the spin structure of the wire is similar to one of a magnetic monodomain, with gradual in-field reorientation for almost all spins (only the outermost spins at the two planar surfaces of the cylinder exhibit slightly different rotations). This S–W-like behavior in perpendicular geometry does not occur in parallel geometry, as indirectly proven by switching fields that depend on the stiffness constant and are much lower than the saturation field (see inset of Figure 5) and that indicate a magnetization reversal through movement of domain walls. This specific magnetization reversal involving a very fast moving of transversal domains walls (400 nm in about 10^−9^ s) in case of nanowires of 40 nm diameter has been investigated in [33] through time-dependent micromagnetic simulations. Such a fast movement of the domain walls cannot be sensed in a static hysteresis loop, which will change “instantaneously” the direction of the magnetization at the “switching field” similar to the case of a S–W-like coherent rotation. Moreover, there is an equivalent implication in the magnetoresistance behavior, which has to remain constant when passing the applied magnetic field through any vicinity of the switching field (Figure 2).

**Figure 6:**
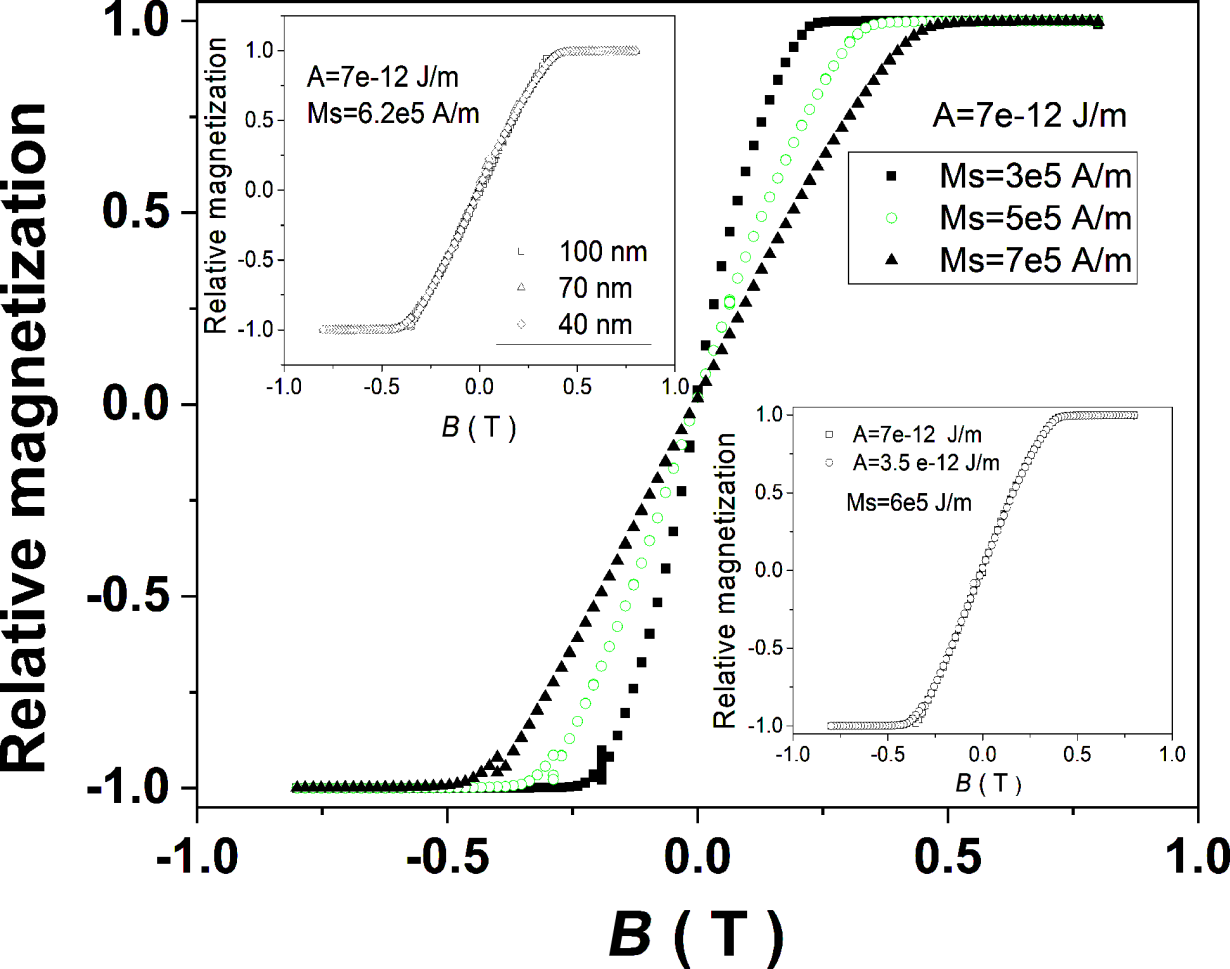
Magnetization reversal in almost perpendicular geometry in Ni–Cu alloy nanowires with diameters of 100 nm and at different realistic values of the stiffness constant and saturation magnetization. The upper inset proves clearly that the S–W-like mechanism in this geometry is active also for nanowires with diameters of 100 nm. The lower inset shows that, similarly to the loops of nanowires with diameters of 40 nm, the hysteresis loops of nanowires with diameters of 100 nm do not depend on the values of the stiffness constant.

In order to support theoretically the mechanism of a S–W-like coherent rotation in transversal geometry also for nanowires with diameters according to the real magnetoresistance experiments, micromagnetic simulations have been performed within the same range of magnetic parameters but with a diameter of 100 nm. The results obtained for *A* = 7·10^−12^ J·m^−1^ and *M*_S_ = 6.2·10^5^ A·m^−1^ are shown in the upper inset of Figure 6, proving clearly that the hysteresis loops are identical for nanowires with diameters of up to 100 nm and consequently the S–W-like rotation is present also in this case. On the other hand, the lower inset in Figure 6 shows almost identical hysteresis loops (and consequently identical saturation fields) for different values of *A*. Hence, only the dependence of the hysteresis loops on *M*_S_ are of interest (Figure 6). In fact, S–W-like rotation mechanisms in perpendicular geometry for nanowires of high aspect ratio with different diameters (even of 700 nm) have been previously assumed (without any theoretical support) for the interpretation of some magnetoresistance data in transversal geometry [34–35].

In the present case, the simulated magnetization reversal in perpendicular geometry (Figure 6) resemble well the results derived from the magnetoresistive curves in perpendicular geometry. Saturation fields close to the experimental values are obtained for saturation magnetization values close to 6·10^5^ A·m^−1^. It is worth mentioning that this value is much larger than the typical value of bulk Ni at 300 K of about 4.8·10^5^ A·m^−1^ with a magnetic moment for Ni of about 0.75μ_B_. This is a surprising result inferring specific electronic structures in Ni nanowires, leading to magnetic moments enhanced by 25% with respect to bulk Ni.

Both saturation and coercive field of the nanowire arrays are much lower than those of single nanowires (with material-related magnetic parameters selected from the best agreement between simulation and experiment). The much lower coercive field in case of the nanowires array (about 0.01 T) as compared to the switching field of a single nanowire (of the order of 0.1 T) in parallel geometry, as well as the much smoother reversal in the first case hint to a progressive rotation of magnetic moments of nanowires antiferromagnetically coupled by magnetic dipolar interactions.

**Figure 7:**
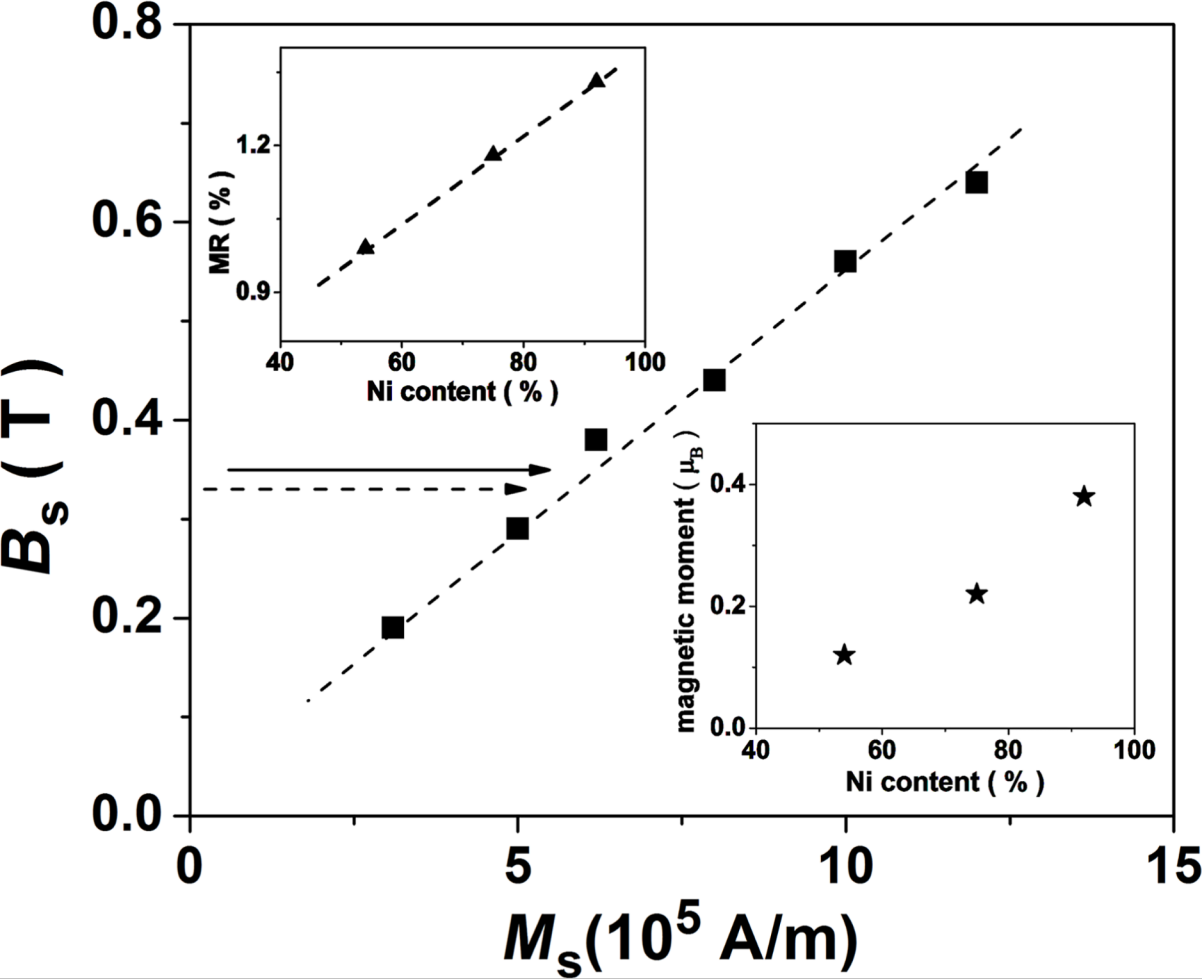
Saturation field strength as a function of the spontaneous magnetization obtained by micromagnetic simulations of a single nanowire. The average magnetic moment of a Ni atom as a function of the Ni content as obtained from magnetization measurements on nanowire arrays is presented in the lower inset. The magnetoresistive effect as a function of the Ni content as obtained from single nanowire measurements is shown in the upper inset.

Surprisingly, the average magnetic moment per Ni atom, as derived from the magnetic loops collected on nanowire arrays, increases with the Ni content whereas the experimental saturation fields corresponding to different single nanowires remain almost constant. The dependence of the saturation field strength on the spontaneous magnetization (or implicitly the magnetic moment of a Ni atom) obtained by micromagnetic simulations is shown in Figure 7.

An almost linear increase of the saturation field as a function of the spontaneous magnetization is observed, as expected in a monodomain-like magnetic system with shape anisotropy. The average magnetic moment of a Ni atom shows an increasing trend with the Ni concentration, indicating an increasing magnetic contribution of Ni in the corresponding samples (see lower inset in Figure 7). If so, the only explanation for the very slight decrease of the saturation field from 0.36 T in sample SNW3 (92% Ni) to only 0.34 T in sample SNW1 (54% Ni) is an enhanced segregation of Ni and Cu, with the formation of a segmented structure consisting of Cu and Ni volumes. The Ni-enriched cylindrical segments still exhibit the high aspect ratio required for shape anisotropy and hence, their lengths of hundreds of nanometers are consistent with anisotropic magnetoresistive effects and not with a GMR spin-valve configuration of a segmented structure with magnetic/nonmagnetic layers of nanometer thickness. In this case, an increased average saturation magnetization per wire can be obtained through higher Ni contents, while the local magnetoresistive behavior has to be related to each Ni-enriched cylinder. However, the fact that the magnetoresistive effect increases slightly with the average Ni content in the samples (from about 1.0% in SNW1 to 1.4% in SNW2) suggests more pure Ni volumes at increased Ni concentrations. According to [26], the magnetoresistance in Ni–Cu alloys (in the Ni-rich concentration range) increases by about 20% with each Bohr magneton. Hence, an increased MR effect by only 0.4% would mean an increase of the Ni magnetic moment by 0.02µ_B_ or an equivalent increase of 3% in the saturation magnetization of 6·10^5^ A·m^−1^. This leads to a variation of less than 0.02 T in the saturation field (Figure 7), as experimentally obtained in this work.

## Conclusion

Ni–Cu alloy nanowires of different Ni content were grown using electrodeposition in polycarbonate nanoporous membranes. Contacting single Ni–Cu alloy nanowires by means of EBL allowed magnetoresistance measurements to be made on individual single Ni–Cu alloy nanowires with diameters of 100 nm. Magnetoresitive effects of the order of percent were observed in perpendicular geometry for all investigated samples and interpreted in terms of anisotropic magnetoresistance. Within this interpretation, a direct simple methodology for the transformation of the magnetoresistance data in transversal geometry into the corresponding hysteresis loops of single nanowires was proposed. This simple strategy is based on time-independent micromagnetic calculations of Ni–Cu nanowires of high aspect ratio with realistic magnetic parameters and different diameters (up to 100 nm). Specific magnetic parameters of single nanowires with different Ni content were derived by the correlation of micromagnetic simulations adapted to such nanostructures and the experimental magnetic hysteresis loops provided by the magnetoresistance data. A segment-like growing of the nanowires with Ni and Cu enriched segments (as compared to average alloy compositions) as well as an unexpected high value for the magnetic moment of Ni in the as-grown nanowires (0.75 μ_B_ compared to 0.61μ_B_ in pure bulk Ni) have been shown.

## Experimental

### Preparation of Ni–Cu alloy nanowires

Ni–Cu alloy nanowires were prepared using a templated electrochemical deposition/replication approach. The templates used were nanoporous polycarbonate membranes with a thickness of 30 µm, a density of 10^9^ pores·cm^−1^ and a pore diameter of 130 nm. Chemical etching of the pores was performed by immersing the irradiated polycarbonate foils in an aqueous solution of 5 M NaOH and 10 vol % methanol at a temperature of 50 °C. The working electrode, a thin film (50 nm) of Au, was sputtered on one side of the polycarbonate membrane and was subsequently electrochemically thickened with a thin layer of Cu (10 µm).

The electrodeposition was made using a modified Watts bath containing: 225 g·L^−1^ NiSO_4_, 2 g·L^−1^ CuSO_4_, 30 g·L^−1^ NiCl_2_, 32.5 g·L^−1^ boric acid H_3_BO_3_ and 5 g·L^−1^ PVP. All chemicals were acquired from Sigma-Aldrich and used as received. The concentration of copper ions in the bath was about 100 times lower than the concentration of Ni ions since Cu can be reduced at less electronegative working electrode potential than Ni.

Electrodeposition was performed using a potentiostat/galvanostat (PARSTAT 2276) controlled by a PC and employing a typical three-electrode configuration: a platinum foil of 1 cm^2^ (counter electrode), a thin film of Au covering one side of the polycarbonate membrane (working electrode) and a commercial saturated calomel electrode (SCE) as a reference electrode. The process temperature was kept constant at 50 °C by using a double-wall electrochemical cell and a recirculating bath. The nanowires were synthesized at four different electrodeposition potentials: −800 mV, −900 mV, −1000 mV and −1050 mV in order to have different compositions, because the ratio between Ni and Cu changes depending on the potential.

### Contacting single Ni–Cu alloy nanowires

In order to investigate the galvanomagnetic properties of single Ni–Cu alloy nanowires, first the template was dissolved successively in chloroform and dichloromethane. Further, by ultrasonication, the nanowires were transferred in ultrapure isopropanol and placed on n^++^-doped SiO_2_/Si substrates having interdigitated metallic electrodes of Ti/Au (10/90 nm). The electrodes were obtained combining photolithography (using an EVG 620 equipment situated into a cleanroom class 100 (ISO EN 14644) with RF magnetron sputtering and thermal vacuum evaporation (using TECTRA equipment). Thus, to contact single Ni–Cu alloy nanowires using a typical EBL process (Zeiss Merlin Compact field-emission scanning electron microscope combined with a Raith system), a droplet of nanowires suspension in isopropanol is placed on the substrate with interdigitated metallic electrodes of Ti/Au.

**Figure 8:**
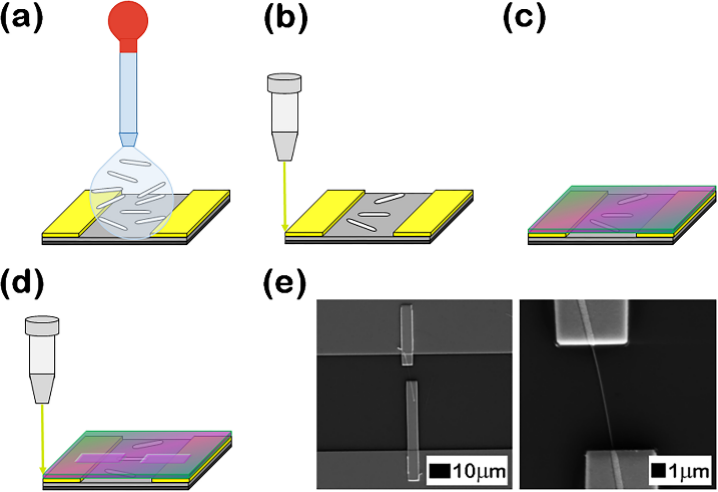
(a) Ni–Cu alloy nanowires placed on SiO2/Si substrates between the interdigitated metallic electrodes obtained by photolithography; (b) alignment of the SiO_2_/Si substrate with the sample holder of the microscope; (c) the sample covered with a poly(methyl methacrylate) film deposited by spin coating; (d) irradiation of the polymer layer for contacting the ends of the nanowire with the interdigitated metallic electrode; (e) SEM images of a single Ni–Cu alloy nanowire contacted using EBL.

Individual nanowires conveniently placed between the photolithographically fabricated electrodes were chosen for contacting. After the specific EBL process, the deposition of Ti and Au contacts (100/200 nm) by RF magnetron sputtering and thermal vacuum evaporation, respectively, was performed. Individual contacted Ni–Cu alloy nanowires are thus obtained. The main steps of the EBL process are shown in Figure 8.

The morphological and compositional properties of the nanowires were investigated by means of scanning electron microscopy and energy-dispersive X-ray spectroscopy using a Zeiss Evo 50 XVP scanning electron microscope (SEM) with an energy dispersive X-ray analysis (EDX) Quantax Bruker 200 as accessory.

Magnetoresistance measurements were made using a system composed of a closed-cycle He cryostat (Janis SHI-4ST-1; 4–300K), LakeShore electromagnet EM4-HVA (2.5 T) with power supply 642 (0–70 A, 35 V), temperature controller LakeShore 331S, LakeShore DSP 475 Gauss meter, Keithley 2612A current/voltage source, Keithley 6517A multimeter and vacuum system HiCube Pfeiffer (10^−6^ Torr).

Micromagnetic simulations have been made using the free software package OOMMF (Object Oriented Micromagnetic Framework) by NIST [36].

## Supporting Information

File 1 Additional experimental data.
