# Supplementary material for: Magnetism and magnetoresistance of single Ni–Cu alloy nanowires
Source: Beilstein J Nanotechnol. 2018 Aug 30;9:2345–55. doi: 10.3762/bjnano.9.219 (PMC6122149; doi:10.3762/bjnano.9.219)
Supplement: File 1 — Additional experimental data. [file Beilstein_J_Nanotechnol-09-2345-s001.pdf]

Supporting Information

for

Magnetism and magnetoresistance of single Ni–Cu alloy nanowires

Andreea Costas[1], Camelia Florica[1], Elena Matei[1], Maria Eugenia Toimil-Molares[2], Ionel Stavarache[1], Andrei Kuncser[1,3], Victor Kuncser*[1] and Ionut Enculescu*[1]

Address: [1]National Institute of Materials Physics, PO Box MG-7, 077125, Magurele-Bucharest, Romania; [2]GSI, Helmholtz Centre, Planck str. 1, D-64291, Darmstadt, Germany and [3]University of Bucharest, Faculty of Physics, PO Box MG-11, 077125, Magurele-Bucharest, Romania

Email: Victor Kuncser* - kuncser@infim.ro; Ionut Enculescu*: encu@infim.ro

* Corresponding author

Additional experimental data

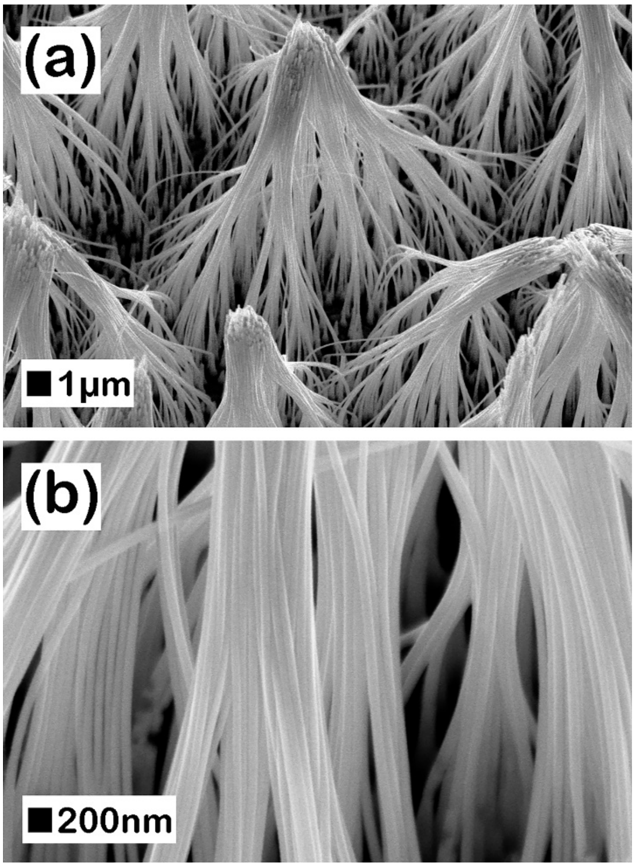

Figure S1: SEM images at different magnifications for arrays of Ni–Cu alloy nanowires grown by electrochemical deposition at −1000 mV in nanoporous polycarbonate membranes.

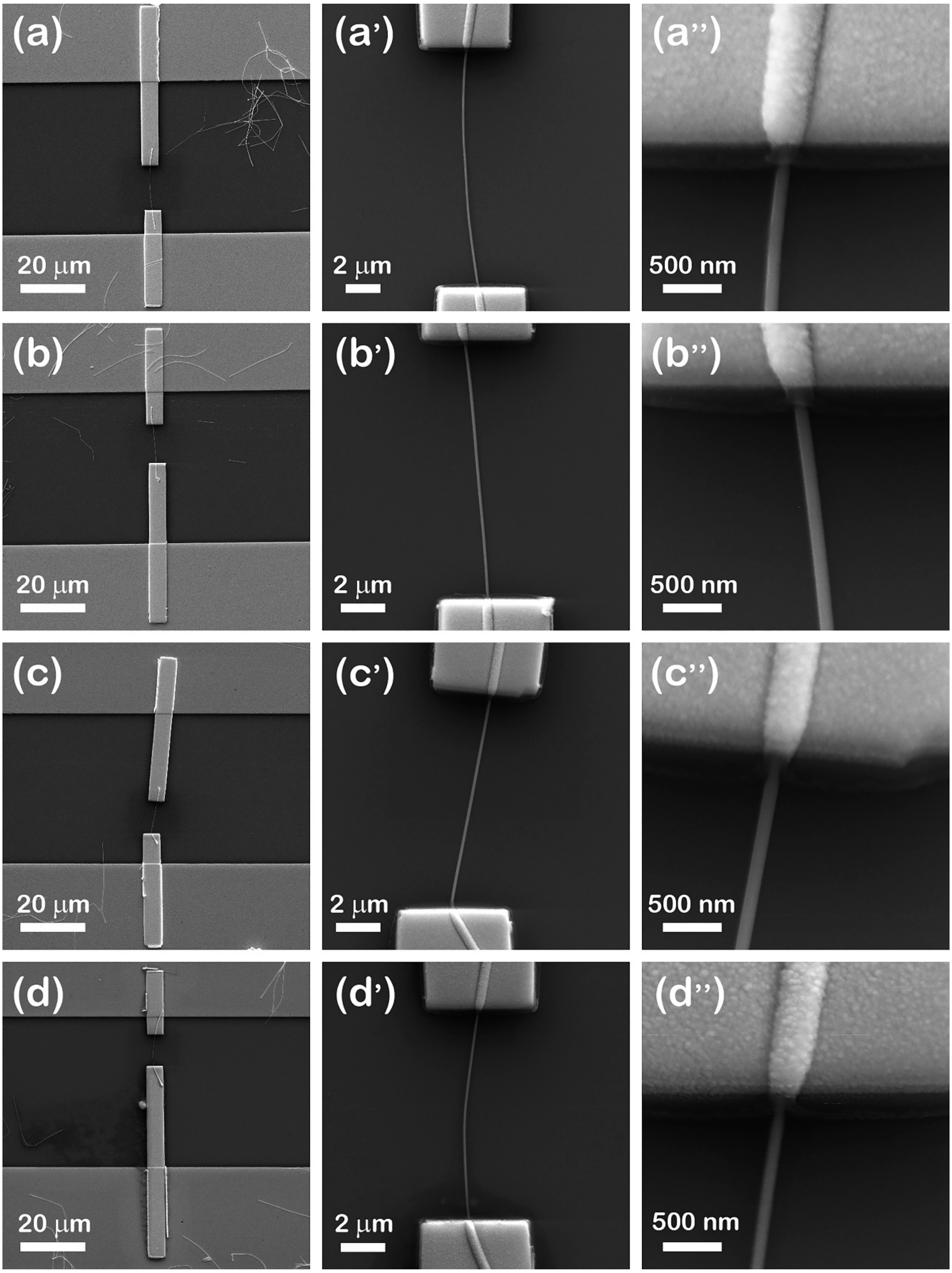

Figure S2: SEM images of Ni–Cu alloy nanowires contacted using EBL, grown by electrochemical deposition at different electrodeposition potentials: (a, a′, a″) −800 mV; (b, b′, b″) −900 mV; (c, c′, c″) −1000 mV and (d, d′, d″) −1050 mV.

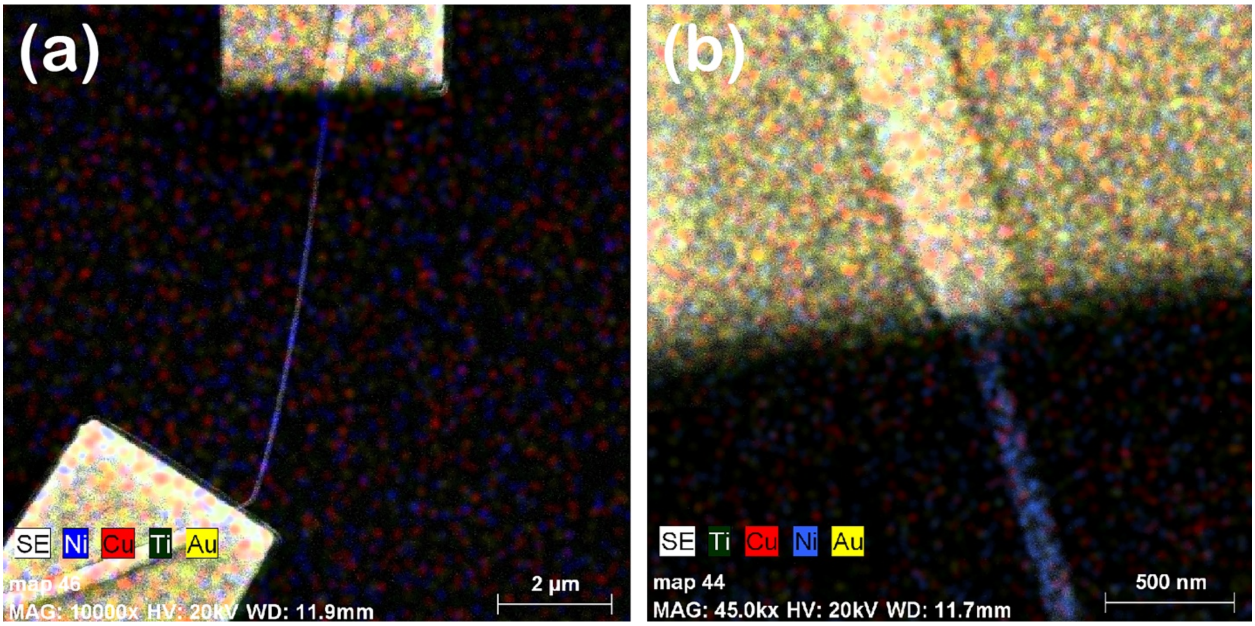

Figure S3: EDX analysis of the distribution of elements in two Ni–Cu alloy nanowires obtained at: (a) −1000 mV and (b) −1050 mV, contacted by EBL with Ti/Au (10 nm/90 nm) metallic contacts at the end of the nanowires.
